# Impact of reduced marker set estimation of genomic relationship matrices on genomic selection for feed efficiency in Angus cattle

**DOI:** 10.1186/1471-2156-11-24

**Published:** 2010-04-19

**Authors:** Megan M Rolf, Jeremy F Taylor, Robert D Schnabel, Stephanie D McKay, Matthew C McClure, Sally L Northcutt, Monty S Kerley, Robert L Weaber

**Affiliations:** 1 Division of Animal Sciences, University of Missouri, Columbia, MO 65211-5300, USA; 2 American Angus Association, Saint Joseph, MO 64506, USA

## Abstract

**Background:**

Molecular estimates of breeding value are expected to increase selection response due to improvements in the accuracy of selection and a reduction in generation interval, particularly for traits that are difficult or expensive to record or are measured late in life. Several statistical methods for incorporating molecular data into breeding value estimation have been proposed, however, most studies have utilized simulated data in which the generated linkage disequilibrium may not represent the targeted livestock population. A genomic relationship matrix was developed for 698 Angus steers and 1,707 Angus sires using 41,028 single nucleotide polymorphisms and breeding values were estimated using feed efficiency phenotypes (average daily feed intake, residual feed intake, and average daily gain) recorded on the steers. The number of SNPs needed to accurately estimate a genomic relationship matrix was evaluated in this population.

**Results:**

Results were compared to estimates produced from pedigree-based mixed model analysis of 862 Angus steers with 34,864 identified paternal relatives but no female ancestors. Estimates of additive genetic variance and breeding value accuracies were similar for AFI and RFI using the numerator and genomic relationship matrices despite fewer animals in the genomic analysis. Bootstrap analyses indicated that 2,500-10,000 markers are required for robust estimation of genomic relationship matrices in cattle.

**Conclusions:**

This research shows that breeding values and their accuracies may be estimated for commercially important sires for traits recorded in experimental populations without the need for pedigree data to establish identity by descent between members of the commercial and experimental populations when at least 2,500 SNPs are available for the generation of a genomic relationship matrix.

## Background

The advent of national genetic evaluation in beef cattle was made possible by the formulation of best linear unbiased prediction (BLUP) via the mixed model equations [1] and most livestock species now use BLUP for the evaluation of additive genetic merit and selection of parents to produce the next generation of progeny. However, most traits for which estimated breeding values or expected progeny differences (EPDs) are computed measure animal outputs rather than inputs. Because of the increased cost of production system inputs, interest has recently been stimulated for the development of efficient methods for producing phenotypes and EPDs for the efficiency of feed utilization. Feed costs in calf feeding and yearling finishing systems account for approximately 66% and 77% of total costs, respectively [2] and while increasing growth rate by 10% has been estimated to increase profitability by 18%, increasing the efficiency of growth of feedlot cattle by 10% is expected to increase profitability by 43% [3]. Other studies have suggested that increasing the feed efficiency of feedlot cattle has seven to eight times the economic impact of similar increases in growth [4]. Selection to improve feed efficiency in cattle has been difficult to accomplish [5] and little progress has been made. Furthermore, guidelines have not yet been created to define the optimal trait upon which to practice selection. While early research focused on growth rate [6], unfavorable correlated responses in other traits, such as mature size, result in economic penalties in other sectors of the production system [4,5]. The popularity of residual feed intake (RFI), or net feed intake, first proposed by KOCH *et al*. [6] as a measure of feed efficiency, is increasing. RFI is phenotypically independent of growth rate and metabolic body weight and can, if desired, be forced to be independent of other factors such as body composition. However, phenotypic independence does not guarantee genetic independence between RFI and the traits upon which it has been conditioned [7] and undesirable correlated responses can occur if producers fail to select on appropriate indexes. RFI also requires the routine and accurate collection of average daily feed intake (AFI) data on large numbers of individuals. Because AFI can relatively easily be assigned an economic value, unlike RFI [8], it is the most logical input trait to include in a selection index [9] which also includes economically relevant output traits, to produce the optimal selection tool [10].

The cost and logistical difficulty of collecting feed intake data on large numbers of animals necessitates the consideration of alternative approaches to the estimation of EPDs for this trait and the application of genomic information is very appealing. While marker assisted selection could be employed, the approach explains only a small portion of the genetic variation within a trait and neglects the variation due to quantitative trait loci (QTL) with small effects for which markers have not been identified [11,12]. Conversely, Genomic Selection (GS) is an option which allows simultaneous selection on all of the QTL that underlie a trait. GS constructs prediction models for EPDs using a training population that possesses phenotypes or EPDs and is genotyped at high density using tens, or hundreds, of thousands of markers. Key to the approach is to calibrate the number of markers that are scored to the extent of linkage disequilibrium (LD) that is present in the genome of the species. By genotyping an appropriately large number of evenly spaced markers which span the entire genome, most QTL are expected to be in LD with at least some of the markers [13]. Provided the training population is appropriately large, GS prediction models can greatly increase the accuracy of EPDs for traits on which phenotypes are especially difficult or expensive to collect. The improvement in selection response due to the application of GS has been estimated to be twice that of traditional selection schemes due to dramatic reductions in generation interval [14] and increases in selection intensity [15,16].

The U.S. dairy industry has aggressively developed systems to utilize genomic information in animal selection, and has provided a model for implementation of GS in the beef industry. Several methods have been proposed for the design of GS programs within dairy breeding programs, primarily using simulated data [15,17-21], although it has not been clear that the applied marker densities have been calibrated to the LD present within the simulated populations. Three methods proposed for the estimation of molecular breeding values include the estimation and summation of individual allele or haplotype effects across all marker loci [15,17,21,22], the replacement of the pedigree-derived numerator relationship matrix with a genomic relationship matrix (GRM) in traditional mixed models [14,16,19-21,23-25] or a hybrid approach involving the use of a GRM to estimate EPDs which are then combined with traditional BLUP of EPDs in a selection index [14,16,21]. The multiple trait derivative free restricted maximum likelihood (MTDFREML) [26] software has been modified to facilitate the prediction of breeding values using GRMs [27]. While GS is now being tested within commercial dairy cattle populations [14,28,29], traditional progeny testing schemes used to achieve high accuracies on the bulls released for widespread use are being modified to reflect the gains in selection response that are possible when bulls, at birth, have estimates of genetic merit with accuracies that are similar to those achieved, on average, with 11 daughter equivalents [14].

The objective of this study was to evaluate the use of GRMs within mixed linear model analyses for variance component estimation, the correction of observations for fixed effects, and for the estimation of molecular breeding values for commercially important sires for traits recorded in experimental populations with incomplete pedigree data using feed efficiency traits as an example. The number of SNP markers needed for accurate generation of a GRM was also explored.

## Methods

### Population Structure

Individual animal feed intake records including average daily feed intake (AFI), residual feed intake (RFI) and average daily gain (ADG), all measured in units of kg/d, were obtained on 862 parent identified Angus steers born between 1998 and 2005 at the Circle A Angus Ranch (Iberia, Stockton and Huntsville, MO; n = 653) and research farms participating in the MFA Inc. feeding trials (Thompson and Greenley, MO; n = 209). All animals were individually fed using commercial feedlot rations either at the Circle A Ranch in Iberia (using Calan gate feeding systems), or at the University of Missouri (using GrowSafe feeding systems). While daily feed intake data were available for all animals, live weights were taken only at the beginning, midpoint and ending of the feeding trial. Descriptive statistics for the collected phenotypes are presented in Table 1. Blood samples (10 mL) were collected on 698 of the steers at the Circle A Ranch on the first weigh date before commencement of the feeding trial. No blood samples were collected during the first year of the feeding trial and of the 862 animals with feed intake records only 698 had DNA available for analysis. The samples were stored in vacuum tubes containing 15 mg of EDTA (Coviden, Mansfield, MA) on ice while being transported. The tubes were centrifuged and the white blood cells removed for DNA extraction. All animal procedures were approved by the University of Missouri Animal Care and Use Committee.

**Table 1:** Descriptive Statistics: Descriptive statistics for three feed efficiency traits and estimates of variance components and heritability from linear model analyses incorporating either numerator or genomic relationship matrices.

Trait^a^	N	Mean	Min	Max	Var			h^2^
**AFI**	862	11.0326	6.0599	15.2116	3.0323	0.1436	0.7786	0.16
**RFI**	862	0.0026	-3.3386	4.9952	0.7626	0.1147	0.4364	0.21
**ADG**	862	1.5363	0.0231	2.3443	0.1077	0.000002	0.552	0.00
**AFI**	698^**b**^	10.8943	6.0599	15.2116	3.1608	0.1404	0.8680	0.14
**RFI**	698^**b**^	-0.0201	-3.3412	4.9952	0.8255	0.0849	0.5286	0.14
**ADG**	698^**b**^	1.5175	0.0231	2.2941	0.1105	0.0053	0.0528	0.09

^**a**^Average daily feed intake, AFI; residual feed intake, RFI; and average daily gain, ADG; all measured in units of kg/d.

^**b**^DNA samples were available on only 698 of the 862 phenotyped steers. Variance components for these three analyses were estimated using the GRM.

Cryopreserved units of semen were also obtained for 1,721 registered Angus sires born between 1956 and 2003 that were used in artificial insemination (AI) within the U.S. Angus population. These animals formed paternal lineages which included the sires of the steer calves and their male ancestors. Genomic DNA was isolated from both the white blood cell and semen samples by proteinase-K digestion followed by phenol:chloroform:isoamyl alcohol extraction, and ethanol precipitation [30]. Additionally, complete pedigrees spanning up to 62 ancestral generations were obtained for the AI sires from the American Angus Association.

Dams of steers were from a population of unregistered commercial purebred Angus cows with pedigree information that was determined to be unreliable based on our attempts to phase chromosomes and infer missing genotypes using linkage information. Because the Circle A Ranch utilized an animal identification system based upon year of birth (two digits) and birth order within each year and the pedigree file did not contain birth date, several alternate pedigrees were possible for many of the steers. Since not all of the putative maternal grandsires had been genotyped with the BovineSNP50 assay, we were unable to correctly identify the maternal pedigree on many of the steers and the parents of each of the dams were treated as unknown for all analyses. However, identifiers for dams were retained to preserve the identification of progeny that were maternal half-sibs. The 862 (698 with DNA) steers had 118 (100) sires and half-sib family sizes ranged from 1 to 81 progeny.

### Data Acquisition

Residual feed intake was calculated as the difference between observed and expected feed intake (), which was predicted from the regression of average daily feed intake on a dry matter intake basis (AFI) on ADG and metabolic midweight (MMW; mid-weight^0.75^) as follows:

Intake and gain data were obtained on feeding groups of 96 steers gathered over a five year period from 1999 to 2003. Cattle were an average of 326 days of age when entering the trial and were fed for an average of 110 days. This feeding period has been found to be sufficient to accurately measure both gain and intake in British breeds [31]. Weights were measured at the start of ration acclimation, on the first day of the test, mid-test and at the end of the test. As these cattle were commercially owned, the specific ration composition is not known, however all animals within a feeding group were fed the same ration. RFI was calculated individually for each feeding group and the mean R^2 ^value for the regression models was 0.49.

SNP genotypes were acquired using the Illumina BovineSNP50 assay [32-34] and genotypes were called using the BeadStudio genotyping module 3.2.32 (ILLUMINA Inc., San Diego, CA). After screening for Mendelian inheritance to verify the accuracy of the sire pedigrees, genotypes for nine of the sires were found to be inconsistent with their paternal pedigree and two were identical twins produced by embryo transfer and these animals were removed from the data set. The genotypes were also filtered to require minor allele frequency (MAF) to be ≥0.05, and call rate to be > 95% which resulted in 41,028 SNP being retained for analysis on 698 steers and 1,707 AI sires. Average MAF for the 41,028 SNP was 0.28 and the average spacing between the 39,971 SNPs assigned to chromosomes (including 487 on BTAX) in the Btau4.0 assembly was 65.73 ± 68.45 kb. Finally, a total of 0.58% of the genotypes in this dataset were missing and these were imputed using fastPHASE [35] with the Btau4.0 positions and the -T10 and -K20 options.

### Additive effects analysis

Pedigree information on 862 Angus steers including their dams and 34,864 identified paternal ancestors was used to generate a numerator relationship matrix (NRM) [1] representing the covariance structure among breeding values of all individuals. Variance components, breeding values and residuals were estimated using the multiple trait derivative free restricted maximum likelihood (MTDFREML) program [[26], VAN VLECK pers. comm.]. Model parameters were estimated iteratively and convergence was assumed when the variance of the -2*log-likelihood was ≤ 1 × 10^-12^. The animal model [36] used for variance component estimation and genetic predictions was:

where: *y *is a vector of phenotypes on the 862 steers, ***X ***is an incidence matrix relating observations to feeding pens, *b *is a vector of pen effects, ***Z ***is an incidence matrix relating observations to animals, *u *is a vector of normally distributed breeding values, and *e *is a vector of independent normally distributed random residuals. The variance of *u *is **A** where **A **is the NRM and  is the additive genetic variance; the variance of *e *is **I** where **I **is the identity matrix and  is the residual variance; *u *and *e *were assumed to be uncorrelated.

Preliminary analyses indicated that birth year (Y), birth season (S) and feeding pen (PEN) were significant sources of variation for almost all traits (AFI, Y p < 0.0001, S p < 0.0006, PEN p < 0.0001; and RFI, Y p < 0.0001, S p < 0.7318, PEN p < 0.0001). However, levels of Y and S were nested within levels of PEN, and PEN was the only fixed effect incorporated into the analysis models.

### Genomic relationship matrix

**Figure 1:**
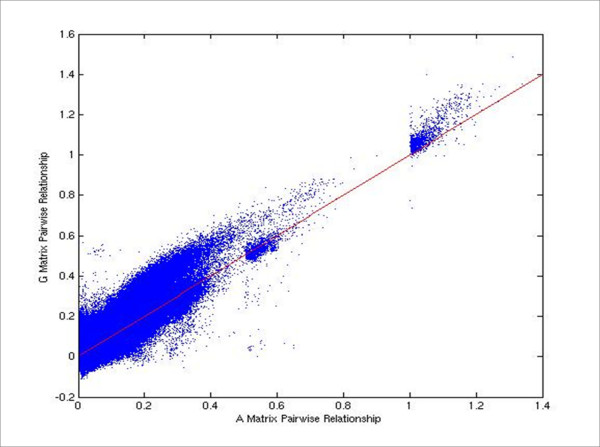
**Plot of GRM vs. NRM matrix coefficients**. Plot of genomic relationship (G) against corresponding additive numerator relationship (A) matrix coefficients for all pairwise combinations among 1,707 Angus AI sires.

**Figure 2:**
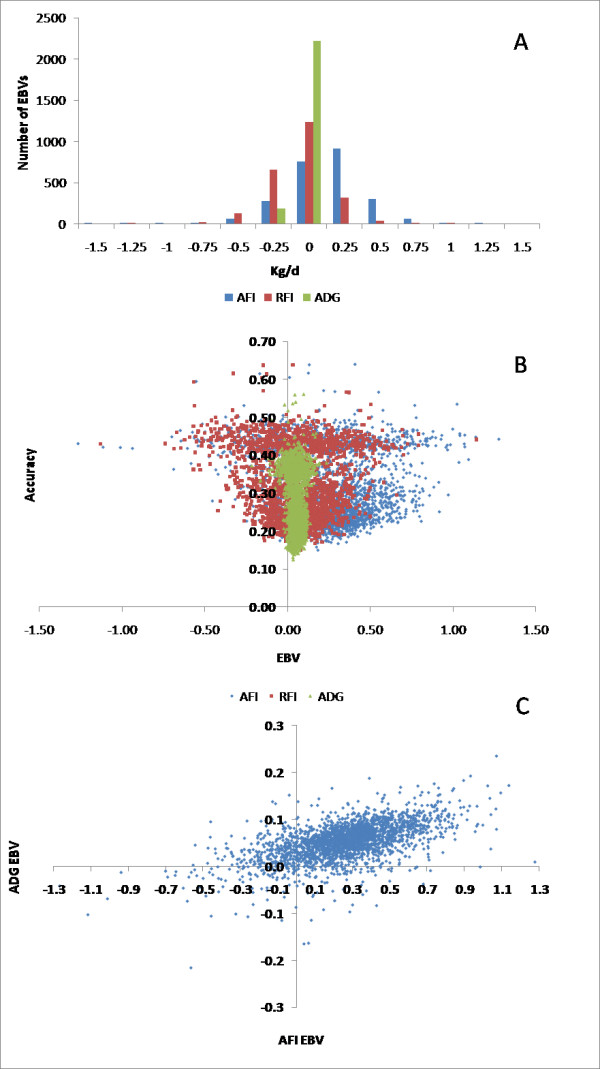
**Estimated breeding value plots using a GRM**. A) Histogram depicting distribution of EBVs. B) Plot of EBVs and their accuracies. C) Plot of AFI versus ADG EBV.

The NRM between the 1,707 sires was generated using the complete pedigree information for these animals [37]. Following imputation of missing genotypes, complete genotypes for 698 Angus steers and 1,707 Angus sires were assembled into a 2,405 × 41,028 genotype matrix (**M**) with animals in rows and SNPs in columns. The matrix **M **contains the elements -1 for *AA*, 0 for *AB *and 1 for *BB *genotypes, respectively [21]. We calibrated the GRM by finding the regression of the upper triangular elements of **MM' **on the corresponding elements of **A **for the 1,707 AI sires and used the estimated slope (*g*_1_)and intercept (*g*_0_) terms to calibrate the GRM for all 2,405 animals as: **G **= [**MM' ***g*_0_(**11'**)]/*g*_1 _[21]. With this approach E[**G**] = **A **when the pedigree information is correct and complete. The method is computationally straightforward and robust [VANRADEN, pers. comm.], and does not require estimation of the SNP allele frequencies within the base population [21]. Our estimates for these parameters in Angus were  = 9,731.9 ± 0.65 and  = 15,198.6 ± 7.26. The average molecular inbreeding coefficient over all 2,405 animals was 0.079. Figure 1 shows a plot of the genomic relationship coefficients against NRM coefficients for all pairwise combinations among the 1,707 AI sires. Variance components, pen effects, breeding values and residuals were estimated under an animal model using restricted maximum likelihood with the GRM used in place of the NRM. The model was assumed to have reached convergence when heritability estimates had converged from above and below to three significant figures. Plots of the estimated breeding values are in Figure 2.

### Construction of Marker Panel Subsets

MATLAB (The Mathworks, Natick, MA) was used to test the number of markers necessary to precisely estimate the GRM by the regression approach in cattle. Subsets (*n *= 100, 500, 1000, 2500, 5000, 10000, 15000, 20000, 25000, 30000, 35000 and 40000) of markers were randomly sampled with replacement from the full set of 41,028 markers to ensure a random representation of the entire genome within the marker subset. For each of *i *= 1,...,50 replicates at each value of *n*, a GRM (**G**_**ni**_) was estimated using the regression approach described above and correlations were estimated between the upper triangular elements of **G**_**ni **_and **G **(the GRM estimated from all 41,028 SNP) for all 2,405 animals and between **G**_**ni **_and **A **for all 1,707 AI sires and averages were produced across replicates.

To simulate the reduced marker panels that are most likely to be commercialized in the beef industry, a panel of 384 SNPs most significant for AFI was selected for the estimation of a GRM. SNPs were first individually screened for their association with AFI using one-way analyses of variance. Subsequently, a chromosome-by-chromosome analysis was performed using a forward selection algorithm in which the SNP with the highest F-statistic for the chromosome was sequentially added to the model until no further SNPs could be added that exceeded a predetermined significance threshold. For this process, the significance threshold was initialized at a genome-wide p-value of 0.05 (F>23.7163) and was relaxed to F>6.33 until a total of 384 SNP were retained for this analysis.

## Results and discussion

Summary statistics presented in Table 1 indicate that there were no appreciable differences between phenotypes for the total sample and for the genotyped subset of animals. Estimates of variance components and of narrow sense heritability using the numerator and genomic relationship matrices are also presented in Table 1. The additive genetic variance components for AFI and RFI were larger when estimated from the full sample using the NRM than when estimated from the subsample using the GRM. However the opposite was true for ADG and rather than reflecting an effect due to sample size, this likely reflects the lack of pedigree information on the dams of these steers which causes them to be treated as unrelated members of the base generation in the analyses that incorporated the NRM. However, the use of the GRM corrects for the identity by descent between these dams, which are all derived from a single herd, and should produce higher allele sharing in their sons than would unrelated females.

Nevertheless, the heritability estimates for all three traits were lower than literature estimates (ADG 0.28 [38]; RFI 0.08-0.44 [5,10]; AFI 0.39 [38]). The reasons underlying the disparity in heritability estimates are unclear, but for ADG and RFI may be due to imprecise estimates of growth, since taking weights at least every 2 weeks during the feeding trial has been recommended [31]. Regardless of the cause, the low heritabilities further reduced the power of this study for the estimation of genomic breeding values.

Breeding values estimated using either the NRM or GRM were strongly correlated for AFI and RFI (0.9074 and 0.9073, respectively) and accuracies estimated as the correlation between true and predicted breeding values [1] were similar (Table 2). Despite the slightly lower heritabilities for AFI and RFI when estimated using the GRM (Table 1) and the fact that there were 164 (19.0%) fewer steers in the GRM analysis, mean accuracies for steers and their sires were similar (Table 2). Presumably, this reflects the ability of the GRM to extract identity by descent information among the steers that was due to the relationships among their dams which were assumed unrelated in the NRM analyses. Accuracies for the GRM analyses were lower than previously reported estimates [15-17,21] reflecting the small sample size and the low heritability estimates obtained in this study. Despite the fact that the heritability estimates for feed efficiency traits obtained in this study were lower than literature estimates, we produced molecular estimates of breeding value for 1,707 bulls which have been among the most influential animals within the U.S. Angus breed. While the accuracies for these estimates are quite low, it should be possible to combine data from multiple sources to perform joint analyses which incorporate data from other Angus-based research populations [38,39] to increase the accuracies of estimated breeding values on current and future AI sires to initiate selection to improve feed efficiency within commercial beef production systems. Even estimates with a low accuracy would allow progress in selection towards efficient conversion of feed until more data can be gathered to improve the genomic estimates of breeding value.

**Table 2:** Accuracies of EBVs estimated using either a NRM or GRM: Average accuracies of estimated breeding value for three feed efficiency traits estimated using mixed linear animal models incorporating either additive numerator (NRM) or genomic relationship matrices (GRM).

Population	Analysis	Number	AFI	RFI	ADG
Steers	GRM	698	0.43	0.43	0.36
	NRM	862	0.40	0.46	-

Sires of Steers	GRM	85	0.44	0.44	0.37
	NRM	100	0.41	0.45	-

AI Sires Pedigree	GRM	1,707	0.27	0.27	0.23
	NRM	34,864	0.01	0.01	-

Total	GRM	2,405	0.32	0.32	0.27
	NRM	35,726	0.02	0.02	-

**Figure 3:**
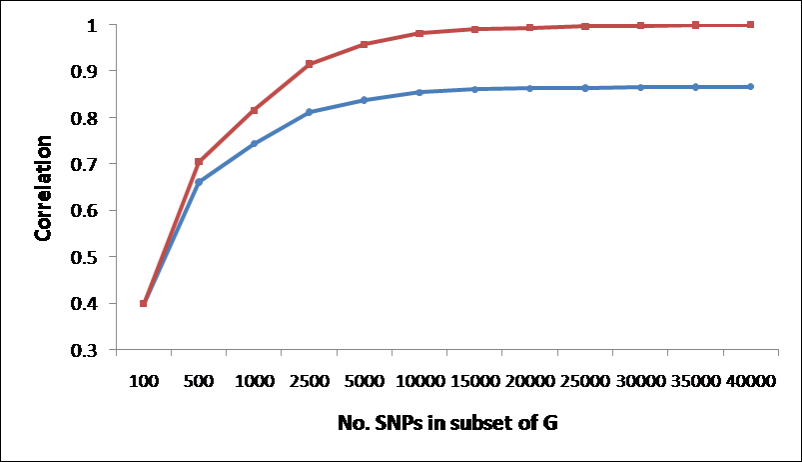
**Bootstrap analysis**. Bootstrap analysis correlations between GRM estimated from subsets of SNPs (G_ni_) and the complete dataset of 41,028 SNPs. Average correlations between G_ni _and NRM (blue) and GRM (red) coefficients with GRM computed from SNP subsets of size n (X-axis) and the average taken across the i = 1,...,50 bootstrap samples.

While the calculation of genomic relationship coefficients is straightforward, it is not clear how many SNP are required to produce robust estimates of relatedness. Figure 3 and Table 3 shows the correlation between upper triangular elements of the GRM computed using all 41,028 SNPs and GRMs computed with subsets of SNPs when averaged across 50 bootstrap replicates for all 2,405 animals within this population. The correlations between upper triangular elements of the GRM computed using subsets of SNPs and the NRM coefficients of 1,707 AI sires with extensive pedigree records are also included. GRM estimated with at least 10,000 markers were highly correlated with the GRM estimated from the complete SNP set (minimum correlation 0.9798) and the mean correlation with the NRM exceeded 0.85. However, the correlation between the NRM and GRM estimated from all 41,028 SNPs was lower than expected and this could be due to several factors. While the sire-son relationships among the 1,721 bulls were validated from their genotypes and only nine inconsistencies were detected in the 29 distinct pedigree generations represented in these data, these may have been due to laboratory errors in DNA extraction, errors in the packaging of semen by a bull stud, or pedigree errors. We suspect that higher rates of pedigree errors may occur in the identification of dams and in the older animals within the pedigree since blood typing of cattle for parentage verification began on a limited basis only in 1940. A second contributing factor could also be the violation, by gametic selection, of the assumption that the Mendelian sampling of parental gametes is independent and has a mean of zero which is required for the computation of the NRM. For example, over the last 20 years, breeders of Angus cattle have selected to improve post-natal growth rate while not changing birth weight. They have accomplished this by selecting the progeny from among matings of selected parents that have intermediate birth weights but high subsequent growth to go on to be the sires extensively used in AI. This form of selection is not modeled in the computation of the NRM and may result in less similarity at the level of the genome than expected based upon pedigree relationship.

**Table 3:** Bootstrap analysis: Correlations between the upper triangular elements of GRMs estimated from subsamples of SNPs with the GRM estimated from 41,028 SNPs and with the NRM computed for 1,707 AI sires with extensive pedigree records.

No. SNPs (*n*)	Correlation between elements of G_ni _and A			Correlation between elements of G_ni _and G		
	
	Min	Mean^a^	Max	Min	Mean^a^	Max
100	0.2773	0.3987	0.4458	0.3545	0.3993	0.4372
500	0.5870	0.661	0.7041	0.6822	0.7050	0.7251
1,000	0.7861	0.7434	0.7861	0.7857	0.8148	0.8275
2,500	0.7871	0.8114	0.8386	0.9061	0.9147	0.9204
5,000	0.7971	0.8375	0.8554	0.9498	0.9573	0.9610
10,000	0.8398	0.8536	0.8641	0.9798	0.9811	0.9821
15,000	0.8508	0.8605	0.8706	0.9886	0.9893	0.9899
20,000	0.8561	0.8624	0.8711	0.9929	0.9934	0.9939
25,000	0.8576	0.8632	0.8707	0.9955	0.9960	0.9962
30,000	0.8577	0.8648	0.8694	0.9975	0.9977	0.9978
35,000	0.8616	0.8656	0.8687	0.9988	0.9989	0.9990
40,000	0.8647	0.8662	0.8679	0.9998	0.9998	0.9998

^a^Averaged over 50 bootstrap samples

While estimates of genomic relationship coefficients based upon at least 10,000 SNPs appear to be extremely robust, estimates appear to be very sensitive to SNP sample size when fewer than 2,500 SNP are used (Figure 3). This has very significant consequences for both conservation genetic and GS applications because there are currently no cost effective technologies available for genotyping 2,500-10,000 SNP markers. Reagent costs for available high density (≥50 K SNPs) assays are in the range $175-$250 per sample and from there, current genotyping technologies allow the genotyping of 1,536 SNPs for ~$70 or 384 SNPs for ~$16 per sample. Consequently, we bootstrap sampled 200 replicates of 384 and 1,536 randomly sampled SNPs from the 41,028 available SNPs and found minimum, mean and maximum correlations between the reduced sample and full GRMs of 0.6046, 0.6536 and 0.6868 and 0.8465, 0.8690 and 0.8821, respectively. However, the 384 or 1,536 SNP panels likely to be commercialized within the livestock industries will not utilize randomly sampled SNP, but will be based on those SNP subsets that are predicted to explain the greatest amount of genetic variation within a trait or set of traits. The effects of computing the GRM from such selected SNP panels will depend on the distribution of LD and MAF among the loci.

When we estimated the GRM using the 1,536 SNPs with the highest MAF (average MAF = 0.4953), the correlation between genomic relationship coefficients was 0.8394, less than the minimum correlation obtained from 50 replicates of random sampling, suggesting that the sampled loci were more strongly linked than the majority of randomly sampled sets of 1,536 SNPs. To test this hypothesis, the average spacing between markers on the same chromosome was tested for the SNP panel with the highest MAF and also for the 200 bootstrap samples. The average spacing between markers in the high MAF panel was 1.8 Mb, and 298 of these markers were less than 250-kb apart on the same chromosome. Conversely, the average spacing of markers on the same chromosome across the 200 bootstrap samples was 38.69 Mb and their average MAF was 0.29. Thus, the SNP with the highest MAF within the Angus genome are tightly linked and provide less information about the relatedness of animals than a randomly sampled panel of SNPs. Just why this is the case is not clear.

When we estimated the GRM using the 1,536 SNP with the lowest MAF (average MAF = 0.0614) the correlation between genomic relationship coefficients was 0.4802. However, when we sampled the 384 SNPs with the highest and lowest MAF (average 0.4980 and 0.0527, respectively), the correlations between genomic relationship coefficients estimated using 384 and 41,028 SNPs were 0.6947 and 0.2226, respectively. The former exceeds the largest correlation obtained in 50 replicate random samples of 384 SNP suggesting that by reducing the number of SNP from 1,536 to 384, the pattern of LD among the 384 loci with the highest MAF is not significantly different to that among randomly sampled loci. Finally, using a forward selection process we identified a panel of 384 SNPs that were most strongly associated with AFI and that had an average MAF of 0.2884. The correlation between genomic relationship coefficients estimated using this sample and the complete set of 41,028 SNPs was 0.6198, slightly lower than the average for randomly sampled SNPs.

These results suggest that the small panels of SNPs that are soon likely to be commercialized within the beef and dairy cattle industries will have some utility for the estimation of genomic relationship coefficients and that this will allow the estimation of molecular breeding values for traits other than those targeted by the SNPs within the panels. However, our results also indicate that the greatest benefits of the technology will not be realized until inexpensive assays can be produced which query ≥2,500 SNPs. When smaller panels of 60- 90 SNPs are used for parentage identification a NRM could be constructed based on the inferred pedigree [40]. We found a correlation between elements of the NRM and GRM based on 41,028 SNPs to be 0.8663, equivalent to the estimation of the GRM with 1,536 randomly sampled SNPs. Thus, the greatest utility from the use of small SNP panels may be the estimation of pedigree to correctly establish the parents of calves since the rates of misidentified parents in the U.S. beef and dairy industries are in the range 3-30% [41].

## Conclusions

Using a genomic relationship matrix, breeding values and their accuracies may be estimated for commercially important sires for traits recorded in experimental populations without the need for pedigree data to establish identity by descent between members of the commercial and experimental populations. This matrix should ideally consist of at least 2,500 SNP in cattle populations, preferably those that are unlinked and not in extremely high linkage disequilibrium with one another. While sufficient numbers of SNPs are not yet available for all species to allow the precise estimation of genomic relationship coefficients, there are no technical limits to rapid and inexpensive SNP development using the deep sequencing of reduced representation libraries with next generation sequencing platforms [32,33]. The most significant limitation to be overcome before the approach will have widespread impact within conservation genetics and livestock improvement communities is the development of inexpensive assays which can simultaneously query from 2,500 to 10,000 SNPs. The number of SNPs available in this population was more than sufficient to generate an accurate GRM, thus the methods applied in this study appear to be viable for the generation of genomic breeding values for feed efficiency traits despite low estimated trait heritabilities. Genomic breeding values for AFI, ADG and RFI were generated for 1,707 Angus AI sires using information on 698 steer progeny from commercial dams with missing pedigree data. These EBVs and accuracies were similar to those obtained from analyses using a NRM despite a 19% difference in the number of animals with phenotypic data. Pooling available data sets on Angus animals should increase the heritability and accuracy of genomic breeding values for feed efficiency traits.

## Authors' contributions

MMR helped conceive the study and its design, performed statistical analysis, assisted in collecting samples, drafted the manuscript and assisted in sample preparation and data collection, JFT helped conceive the study and its design, performed statistical analysis, assisted in collecting samples and helped draft the manuscript, RDS helped conceive the study and its design and assisted in sample preparation and data collection, SDM assisted in sample preparation and data collection, MCM assisted in sample preparation and data collection, SLN assisted in sample and data collection, MSK helped gather data and assisted in phenotype calculations, RLW helped conceive the study and its design, helped perform statistical analysis and helped draft the manuscript. All authors read and approved the final manuscript.

## References

[B1] Henderson CR Best Linear Unbiased Estimation and Prediction under a selection model Biometrics 1975 31 423 447 10.2307/2529430 1174616

[B2] Anderson RV Rasby RJ Klopfenstein TJ Clark RT An evaluation of production and economic efficiency of two beef systems from calving to slaughter J Anim Sci 2005 83 694 704 15705767 10.2527/2005.833694x

[B3] Fox DG Tedeschi LO Guiroy PJ Determining feed intake and feed efficiency of individual cattle fed in groups Proc Beef Impr Fed 33rd Ann Res Symp Annu Meet 2001 33 80 98

[B4] Okine EK Basarab J Goonewardene LA Mir P Residual feed intake and feed efficiency: Difference and implications Florida Ruminant Nutrition Symposium 2004 27 38

[B5] Archer JA Richardson EC Herd RM Arthur PF Potential for selection to improve efficiency of feed use in beef cattle: a review Aust J Exp Agric 1999 50 147 161

[B6] Koch RM Swiger LA Chambers D Gregory KE Efficiency of feed use in beef cattle J Anim Sci 1963 22 486 494

[B7] Kennedy BW Werf JH Van Der Meuwissen TH Genetic and statistical properties of residual feed intake J Anim Sci 1993 71 3239 3250 8294275 10.2527/1993.71123239x

[B8] Garrick DJ Development of genetic evaluations and decision support to improve feed efficiency Proc Beef Impr Fed 38th Ann Res Symp Annu Meet 2006 38 32 40

[B9] Hazel LN The genetic basis for constructing selection indexes Genetics 1943 28 476 490 17247099 10.1093/genetics/28.6.476 PMC1209225

[B10] Herd RM Archer JA Arthur PF Reducing the cost of beef production through genetic improvement in residual feed intake: Opportunity and challenges to application J Anim Sci 2003 81 E9 17

[B11] Meuwissen TH Genomic Selection: The future of animal breeding 2007 88 91 http://www.umb.no/statisk/husdyrforsoksmoter/2007/23.pdf

[B12] Spangler ML Bertrand JK Rekaya R Combining genetic test information and correlated phenotypic records for breeding value estimation J Anim Sci 2007 85 641 649 10.2527/jas.2006-617 17085722

[B13] Meuwissen TH Genomic selection: marker assisted selection on a genome wide scale J Anim Breed Genet 2007 124 321 322 18076468 10.1111/j.1439-0388.2007.00708.x

[B14] VanRaden PM Van Tassell CP Wiggans GR Sonstegard TS Schnabel RD Taylor JF Schenkel FS Invited Review: Reliability of genomic predictions for North American Holstein bulls J Dairy Sci 2009 92 16 24 10.3168/jds.2008-1514 19109259

[B15] Schaeffer LR Strategy for applying genome-wide selection in dairy cattle J Anim Breed Genet 2006 123 218 223 10.1111/j.1439-0388.2006.00595.x 16882088

[B16] Hayes BJ Bowman PJ Chamberlain AJ Goddard ME Invited Review: Genomic selection in dairy cattle: Progress and challenges J Dairy Sci 2009 92 433 443 10.3168/jds.2008-1646 19164653

[B17] Meuwissen TH Hayes BJ Goddard ME Prediction of total genetic value using genome-wide dense marker maps Genetics 2001 157 1819 1829 11290733 10.1093/genetics/157.4.1819 PMC1461589

[B18] Dekkers JCM Prediction of response to marker-assisted and genomic selection using selection index theory J Anim Breed Genet 2007 124 331 341 18076470 10.1111/j.1439-0388.2007.00701.x

[B19] Garrick DJ Equivalent mixed model equations for genomic selection [abstract] J Dairy Sci 2007 90 376 10.3168/jds.S0022-0302(07)72639-5 17183106

[B20] Hayes BJ Goddard ME Technical note: Prediction of breeding values using marker derived relationship matrices J. Anim Sci 2008 86 2089 2092 10.2527/jas.2007-0733 18407982

[B21] VanRaden PM Efficient Methods to Compute Genomic Predictions J Dairy Sci 2008 91 4414 4423 10.3168/jds.2007-0980 18946147

[B22] Calus MPL Meuwissen THE De Roos APW Veerkamp RF Accuracy of genomic selection using different methods to define haplotypes Genetics 2008 178 553 561 10.1534/genetics.107.080838 18202394 PMC2206101

[B23] Van-Arendonk JA Tier MB Kinghorn BP Use of multiple genetic markers in prediction of breeding values Genetics 1994 137 319 329 8056319 10.1093/genetics/137.1.319 PMC1205948

[B24] Matsuda H Iwaisaki H A recursive procedure to compute the gametic relationship matrix and its inverse for marked QTL clusters Genes Genet Syst 2002 77 123 130 10.1266/ggs.77.123 12087195

[B25] Hayes BJ Visscher PM Goddard ME Increased accuracy of artificial selection by using the realized relationship matrix Genet Res, Camb 2009 91 47 60 10.1017/S0016672308009981 19220931

[B26] Boldman KG Kriese LA Van Vleck LD Van Tassell CP Kachman SD A manual for use of MTDFREML. A set of programs to obtain estimates of variance and covariance 1995 USDA, Agriculture Research Service, Clay Center, NE

[B27] Zhang Z Todhunter RJ Buckler ES Van Vleck LD Technical note: Use of marker-based relationships with multiple-trait derivative-free restricted maximal likelihood J Anim Sci 2007 85 881 885 10.2527/jas.2006-656 17085728

[B28] De Roos APW Schrooten C Mullaart E Calus MPL Veerkamp RF Breeding value estimation for fat percentage using dense markers on Bos taurus autosome 14 J Dairy Sci 2007 90 4821 4829 10.3168/jds.2007-0158 17881705

[B29] Guillaume F Fritz S Boichard D Druet T Estimation by simulation of the efficiency of the French marker-assisted selection program in dairy cattle Genet Sel Evol 2008 40 91 102 18096117 10.1186/1297-9686-40-1-91 PMC2674921

[B30] Sambrook J Fritsch EF Maniatis T Molecular Cloning: A Laboratory Manual 1989 Plainview: Cold Spring Harbor Laboratory Press

[B31] Archer JA Arthur PF Herd RM Parnell PF Pitchford WS Optimum postweaning test for measurement of growth rate, feed intake, and feed efficiency in British breed cattle Journal of Animal Science 2009 75 2024 32 10.2527/1997.7582024x 9263047

[B32] Van Tassell CP Smith TPL Matukumalli LK Taylor JF Schnabel RD Lawley CT Haudenschild CD Moore SS Warren WC Sonstegard TS SNP discovery and allele frequency estimation by deep sequencing of reduced representation libraries Nat Methods 2008 5 247 252 10.1038/nmeth.1185 18297082

[B33] Matukumalli LK Lawley CT Schnabel RD Taylor JF Allan MF Heaton MP O'Connell J Moore SS Smith TP Sonstegard TS Van Tassell CP Development and characterization of a high density SNP genotyping assay for cattle PLOS One 2009 4 e5350 10.1371/journal.pone.0005350 19390634 PMC2669730

[B34] Steemers FJ Chang W Lee G Barker DL Shen R Gunderson KL Whole-genome genotyping with the single-base extension assay Nat Methods 2006 3 31 33 10.1038/nmeth842 16369550

[B35] Scheet P Stephens M A fast and flexible statistical model for large-scale population genotype data: applications to inferring missing genotypes and haplotypic phase Am J Hum Genet 2006 78 629 644 10.1086/502802 16532393 PMC1424677

[B36] Quaas RL Pollak EJ Mixed Model Methodology for Farm and Ranch Beef Cattle Testing Programs J Anim Sci 1980 51 1277 1287

[B37] Hudson GFS Quaas RL Van Vleck LD Computer algorithm for the recursive method of calculating large numerator relationship matrices J Dairy Sci 1982 65 2018 2022 10.3168/jds.S0022-0302(82)82454-5

[B38] Arthur PF Archer JA Johnston DJ Herd RM Richardson EC Parnell PF Genetic and phenotypic variance and covariance components for feed intake, feed efficiency, and other postweaning traits in Angus cattle J Anim Sci 2001 79 2805 2811 11768108 10.2527/2001.79112805x

[B39] Sherman EL Nkrumah JD Moore SS Whole genome SNP associations with feed intake and feed efficiency in beef cattle J Anim Sci 2010 88 1 16 22 10.2527/jas.2008-1759 19749024

[B40] Weaber RL A simulation study of replacement sire selection and genetic evaluation strategies for large commercial ranches PhD Dissertation 2006 Cornell University, Animal Sciences Department 193

[B41] Heaton MP Harhay GP Bennett GL Stone RT Grosse WM Casas E Keele JW Smith TPL Chitko-McKown CG Laegreid WW Selection and use of SNP markers for animal identification and paternity analysis in U.S. beef cattle Mammalian Genome 2002 13 272 281 10.1007/s00335-001-2146-3 12016516

